# Randomized controlled trial of the Marriage Checkup: Stress outcomes

**DOI:** 10.1111/jmft.12620

**Published:** 2022-12-16

**Authors:** Astrid B. Leth‐Nissen, Hanne N. Fentz, Tea L. Trillingsgaard, Gertraud Stadler

**Affiliations:** ^1^ The Department of Psychology and Behavioral Sciences Aarhus University Aarhus C Denmark; ^2^ Charité—Universitätsmedizin Berlin, CC1 Health & Human Sciences Gender in Medicine Berlin Germany

**Keywords:** brief couple interventions, individual mental health, longitudinal design, Marriage Checkup, perceived stress

## Abstract

Several couple interventions targeting relationship distress also show beneficial effects on individual mental health. Yet, strikingly few studies report effects on perceived stress. This study examined the effects of a brief couple intervention, the Marriage Checkup (MC), on perceived stress. We randomly assigned 231 couples to receive two MCs (Weeks 7 and 51) or to a waitlist control. Survey data were collected at seven time points over 2 years and analyzed using multilevel models. We found no significant between‐group treatment effects on average stress at any time point. However, women, but not men, in the intervention group experienced decreased stress after the second MC (*d* = −0.23) and more women in the intervention group (26.5%) compared with the control group (14.9%) experienced reliable improvements in stress after the second MC. Overall, the MC did not result in main effects on stress but caused temporary reliable change in terms of stress relief for women.

## INTRODUCTION

The prevalence of stress has increased in the past decades and is one of the most common health concerns today. Internationally, a meta‐analysis from 2021 estimated the average worldwide prevalence of stress to be 36.5% (22 studies from around the world, *N* = 110.849 participants; Nochaiwong et al., 2021). In Denmark, the prevalence of a high perceived stress level has increased from around one‐fifth of Danes in 2008 (*N* = 10.022, L. Nielsen et al., 2008) to around one‐third of Danes in 2021, including 17%–31% of men and 23%–52% of women (depending on age), even though the cut‐off for belonging to the category of being “highly stressed” had been raised^1^ (*N* = 170.979, The National Board of Health, “Sundhedsstyrelsen”, 2022). Moreover, experiencing stress in one domain of life usually spills over into other domains, especially an intimate relationship (e.g., Randall & Bodenmann, 2017). Consequently, stress is costly for the stressed individual, the partner, the employers (through missed days of work or sick leaves), and the public health care system (e.g., Milenkovic, 2019).

Intimate relationships are among the top‐rated sources of daily stress, and nearly half (44%) of adults in the United States report that their romantic partner or kids are a *somewhat* or *very significant source* of stress (American Psychological Association, 2015). Also, a strained relationship increases one's vulnerability to external stressors outside of the relationship due to, for example, low emotional support (Karney & Bradbury, 1995; Slatcher & Schoebi, 2017).

The general understanding of stress originates from Lazarus’ transactional model (Lazarus, 1966; Lazarus & Folkman, 1984), according to which one feels “stressed” when available resources (e.g., time or support) are perceived as insufficient to meet the demands of a particular situation (e.g., work tasks). Although stress is not a medical diagnosis, long‐term overload and stress reactions have shown detrimental effects on numerous aspects of physical and mental health (see the review by Larzelere & Jones, 2008; Thoits, 2010).

Couple interventions aimed at improving couples’ mutual coping with external stressors have shown positive effects on both relationship health (e.g., Bodenmann et al., 2006; Falconier et al., 2015; Ledermann et al., 2007) and individual stress and well‐being (e.g., Bodenmann, 2016; Pihet et al., 2007; Schaer et al., 2008). Also, couple interventions targeting relationship distress have shown positive effects on both relationship satisfaction (Doss et al., 2021; Roddy, Walsh, et al., 2020) and individual mental health (e.g., Proulx et al., 2007), but from this field, findings on perceived stress are (with the few exceptions reviewed below) lacking. This study examined the effects of the brief couple intervention, the Marriage Checkup (MC), on perceived stress.

### The MC

The MC (Cordova, 2014) is a brief, low‐cost, and empirically well‐established (Doss et al., 2021) couple intervention that lowers barriers for couples’ help‐seeking. Equivalent to annual dental checkups, the MC promotes regular (e.g., annually) contact with a professional, thereby bridging the gap between prevention and treatment. In the Danish adaptation of the MC (Trillingsgaard et al., 2017), each checkup consists of two joint 90‐min sessions, one assessment session and one feedback session, that are scheduled 1–2 weeks apart. While not framed as therapy, the primary aim of the MC is to foster partner intimacy and acceptance through methods from integrative behavioral couple therapy (IBCT; Christensen et al., 2020), motivational interviewing (W. Miller & Rollnick, 2002), behavioral activation, and relationship health educational elements. Though manualized, the therapist tailors the session to the couple's unique strengths and concerns, thereby identifying the most adequate plan for subsequent caretaking for the specific couple.

The MC has shown positive effects in several studies including American and Danish randomized‐controlled trials (RCTs) that found small to medium effects of two annual checkups on measures of relationship functioning (e.g., Cordova, Fleming, et al., 2014; Trillingsgaard et al., 2016) and on individual depressive symptoms (Cigrang et al., 2022; Gray et al., 2020). Furthermore, a recent RCT found that a subsample of responder couples benefitting from two checkups could maintain most of their improvements across a 5‐year booster session period (Leth‐Nissen et al., 2022). Additionally, the MC has been successfully adapted to different populations, for example, low‐income at‐risk couples (Gordon et al., 2019), same‐sex couples (Minten & Dykeman, 2019), perinatal couples (Darling et al., 2021), transgender couples (pilot study by Minten & Dykeman, 2021), and military couples (Cigrang et al., 2022).

### How the MC could relieve individual stress

No previous study has reported effects of any relationship checkup model on perceived stress (for a meta‐analysis, see Fentz & Trillingsgaard, 2017; Halford et al., 2017) but, as we will outline, the MC seems to have potential for relieving individual stress. Following the biopsychosocial Strengths and Strains Model of Marital Quality and Physical Health (Slatcher & Schoebi, 2017), individual health, including stress, can be improved through two interrelated paths in couple interventions, both of which are incorporated into the MC.

For the first path, the MC can be assumed to relieve stress by targeting stressors within the relationship itself, for example, conflicts, lack of intimacy, and breaches of trust. In support of this assumption, randomized trials on relationship checkup models that reduced relationship distress did, with few exceptions (e.g., Halford et al., 2017), also find beneficial effects on measures of other aspects of individual mental health including mood or depressive symptoms (Gray et al., 2020; L. R. Miller et al., 2013), anxiety (Woodin & O'Leary, 2010), and global psychological distress (Halford et al., 2012). These findings are generally in line with conclusions from the broader field of couple intervention research, in which interventions aimed at reducing couple distress also improved individual mental health (Cooper et al., 2021; Proulx et al., 2007; Roddy, Rhoades, et al., 2020). However, and against the stress relief assumption, there are strikingly few published findings on individual perceived stress, specifically. In fact, among experimental studies of couple intervention targeting relationship health, we found only one study that reported findings on perceived stress. In their RCT, Roddy, Rhoades, et al. (2020) found that couples receiving one of two online couple interventions (OurRelationship or ePREP), both of which targeted relationship functioning, showed improvements on the Perceived Stress Scale (PSS; Cohen's *d* = −0.42) compared with a waitlist control condition. Although these findings are yet to be replicated for couple interventions in general, and for relationship checkup models in particular, they support the assumption that a relatively brief couple intervention, effective in improving relationship satisfaction, may also reduce individual perceived stress.

For the second path, the MC can be assumed to reduce the impact from stressors external to the relationship (e.g., from work, illness, or finance issues) by increasing stress‐buffering resources in the relationship (e.g., stronger social/emotional support or dyadic coping). In general, interdisciplinary research has vastly confirmed that the impact of stressors on individual stress (as well as general health) is reduced when the individual perceives social support, especially from a romantic partner (e.g., Bodenmann & Randall, 2013; Falconier & Kuhn, 2019). In more concrete support of this second path, several couple‐based interventions developed to increase social/emotional support or mutual coping with external sources of stress (typically chronic illness such as cancer, but also everyday stressors) are effective (Bodenmann, 2010; Scott et al., 2004; Thompson, 2021). One example is the Couples Coping Enhancement Training (Bodenmann & Shantinath, 2004) that, in several RCTs, has been shown to improve not only relationship quality, dyadic coping, and couples’ communication but also individual well‐being and symptoms of work‐related stress and burn‐out (Bodenmann, 2016; Schaer et al., 2008). In line with the field of couple‐based coping interventions, the Danish adaptation of the MC does assess each individual partner's level of stress and each partner is asked to detail current sources of external stress (e.g., work stress, illness, financial stress) before the first session. Also, in the first session, the couple prioritizes one or two primary concerns, and the checkup will be tailored to the issue of mutual coping with external stressors if this is made the priority.

In sum, the weight of the current evidence is in favor of a positive hypothesis. Given that the MC has been found to be effective in improving relationship satisfaction and depression and includes external stressors as an aspect of the intervention, it seems likely that the intervention will also be effective in reducing individual stress. Yet, the lack of published findings on perceived stress outside the field of coping‐focused couple interventions leaves open the possibility that such effects are generally absent when interventions have a more implicit focus on coping with external sources of stress.

### The paradox of health

To the best of our knowledge, no previous study has investigated adverse effects of relationship checkups on stress or other unintended reactions. This may be an oversight, since temporary adverse or ambivalent reactions to therapy are relatively common in accompanying the positive treatment effects (e.g., Barlow, 2010; Berk & Parker, 2009). Especially, since the prevalence of stress is increasing in populations worldwide, inadvertent stress reactions to relationship health checkups seem to be a relevant focus of investigation.

In medicine, scholars have described the *paradox of health*: More accurate measures for detecting health issues, improved treatment methods and outcome, and improved objective health status of the population have *not* led to improved subjective experiences of health and well‐being, but rather to higher self‐reported *dissatisfaction* with own health (Barsky & Barsky, 1988). One explanation is that the expectations and standards that we use for evaluating “health” appear to have been raised along with the heightened focus on health in society and media, leading to higher self‐awareness and stricter evaluation of symptoms that can intensify the perception and experience of symptoms as more disturbing (Barsky & Barsky, 1988). A similar paradox exists within the field of psychological treatments, where sociologists and psychologists have characterized the modern culture to include excessive “coachification,” referring to a performance culture where the individual is expected to constantly be working on improvements in every aspect of life, including romantic relationships, regardless of a relatively healthy baseline (Brinkmann, 2014). The MC applies knowledge from the field of relationship science and encourages a more proactive detection and treatment of relationship health issues. It seems to be a fair concern that recurring processes of evaluating one's relationship up against some standard (e.g., the conceptualization of the relationship's strengths and concerns or the educational elements) can induce a more critical attitude, feelings of ambivalence, or inadvertent stress reactions.

If one partner goes through the steps of problem recognition and help‐seeking considerations before the other partner, an experience of being “dragged by one's feet” to a relationship checkup might be stressful (Doss et al., 2003). When signing up to an MC, some participants experience skeptical anticipations, that is, fear of being blamed or criticized by the partner, that the therapist will “take the partner's side,” or that the consultation will “stir up issues” and cause more pain than currently present (Eubanks Fleming & Córdova, 2012). Also, when entering the MC, the self‐disclosure needed for fostering intimacy can be at least temporarily stressful because it involves the risk of being hurt when being vulnerable (Cordova, 2014). We will assume that if the MC evokes stress (especially for first‐time attenders), it will most likely be temporarily (until healthier couple functioning is established) and therefore not damaging, which speaks against the assumption of prolonged stress reactions to this intervention. This assumption is supported by research showing that after an MC, couples generally report more intimacy and satisfaction (as outlined above) and they rate the intervention as relevant, tolerable, and recommendable (Trillingsgaard et al., 2016). Still, since the MC reaches couples from widely different populations and across the full continuum of relational distress, a heightened focus on any unintended reactions seems justified. When recommending and disseminating mental health care services, unintended “side effects” of these interventions should be evaluated (equivalent to traditional efficacy testing in medicine); yet, this is rarely done or reported in efficacy studies of psychotherapy (Barkham et al., 2021).

### The current study

From the outlines in the introduction, there seems to be a potential for the MC to reduce perceived stress as different kinds of couple interventions have been shown to improve individual mental health while aiming at reducing relationship distress (the first path) or strengthen dyadic coping with external stressors (the second path). On the other hand, considering the sparse evidence base for effects of couple interventions on perceived stress specifically, it cannot be ruled out that a checkup model can also evoke some level of unintended perceived stress. In the current RCT, we aimed to investigate the positive and negative effects of two annual MCs on women's and men's perceived stress symptoms including its maintenance at the 2‐year follow‐up.

## METHODS

### Participants

Participants were 231 cohabiting couples recruited from two metropolitan areas of Denmark. The sample's age ranged from 24 to 66 years, with a mean age of 37.4 years (standard deviation [SD] = 6.4) for women and 39.1 years (SD = 6.5) for men. The majority (95.5%) were born in Denmark and had an education of bachelor's degree or above (88% of the women, 84% of the men). More than half of the couples (67.6%) had a total income at or above the Danish average (of year 2013), while 9.3% of the couples had an income half that of the average Danish couple or less (Danish Statistics, 2015). Most couples were opposite sex (99.6%), married (80.3%), and dual employed (71.7%). The average length of the relationship was 12.1 years (SD = 6.5). Inclusion criteria were age older than 18 years, cohabiting with one's partner, and living with at least one child younger than 18 years of age. Participants were excluded if they were attending psychotherapy or using psychotropic medication during the study period.

### Design

This study describes a two‐armed RCT. Couples were recruited nationally via newspapers, flyers, broadcast, and online advertisements. Couples were randomly assigned to either an intervention group receiving two MCs scheduled 1 year apart (*n* = 114) or a control group receiving movie tickets Year 0 and a feedback report Year 1 (*n* = 117). Randomization was administered using sequentially numbered, opaque, sealed envelopes. The intervention was conducted from 2013 through 2016 by six psychologists and independent practice therapists in the two largest cities of Denmark located in each end of the country. Further details about the inclusion and intervention procedure as well as results for relationship outcomes across the first year are described in Trillingsgaard et al. (2016), while the current study reports the effects on perceived stress across the full 2‐year period. The flow of participants is illustrated in Supporting Information: Figure S1. Couples in the intervention group filled in online surveys at Weeks 0 (baseline), 10, 21, 34, 47, 54, and 104. They received two MCs around Weeks 7 and 51. Couples in the control group reported perceived stress at Weeks 0, 10, 54, and 104. All study procedures complied with standards from the regional ethical committee, and the study was approved by the Danish Data Protection Agency.

### Measures

We measured psychological stress using the Danish 10‐item version of the PSS designed to tap the degree to which individuals generally find their everyday lives unpredictable, uncontrollable, and overloaded with items such as, for example, “*In the last week, how often have you felt nervous and* ‘*stressed*’?” or “…*have you felt that you were on top of things*?” (Cohen et al., 1983). Some PSS items measure the *degree* of perceived stress and some items measure the perceived ability to *cope* with the stress. Originally, the PSS measures stress symptoms in the last months, but because some of our datapoints were relatively close in time, we asked participants to rate their perceived stress in the last week instead of month. Answers are rated on a Likert scale of 0 (*Never*) to 4 (*Very often*) points. Positively worded items are reversely scored, and all items are summed (ranging from 0 to 40), with a higher score indicating higher level of perceived stress. The PSS is considered the best practice for measuring psychological stress (Crosswell & Lockwood, 2020), and it has been validated across varieties of cultures and populations in the past four decades (see, e.g., Juárez‐García et al., 2021, for an overview). The PSS has been translated into Danish by a task force of scholars and clinicians from the field of Medicine and Psychology, who collaborated to form consensus about the best translation (Eskildsen et al., 2015), and the scale has been validated in both a large community sample (*N* = 32,374 Danes; M. G. Nielsen et al., 2016) and in a sample of 64 patients with work‐related stress complaints (Eskildsen et al., 2015). The PSS showed good internal reliability/consistency in the current sample, with a Cronbach's *α* of 0.86 for women and 0.83 for men. In a Danish representative sample (*N* = 10,022), the mean perceived stress among Danes cohabiting with a romantic partner was 11.3 (SD = 5.8) for women and 9.9 (SD = 5.5) for men (L. Nielsen et al., 2008). According to these Danish norms, the cut‐off for a “high stress level” (being the upper 20% percentile of the PSS) is ≥17 for women and ≥15 for men.

Therapists’ adherence to the MC manual was measured by coding a random sample of 20% of all checkups using video recordings, as reported in Trillingsgaard et al. (2016). Following a coding manual, therapist behavior was rated from 0 (*Did not adhere to manual*) to 5 (*Completely adhered to manual*) by two independent raters who were master's degree psychology students. Therapist adherence was high (*M* = 4.63) in both checkups, and the interrater reliability was good as the raters agreed within one level on the scale on 90.9% of their scores (Trillingsgaard et al., 2016).

### Data analyses

Data were analyzed in SPSS 27. The data set can be obtained from the third author upon request.

The sociodemographic characteristics of the sample are reported in Trillingsgaard et al. (2016) including age, education, marital status, length of relationship, dual employment, having biological children or children from previous relationships, being born in Denmark, and location for the intervention (east or west of Denmark). Participants did not differ in these characteristics between the two conditions (Trillingsgaard et al., 2016).

To evaluate the effects of the MCs across the 2‐year period on perceived stress, we constructed a dual‐intercept multilevel model following the recommendations of Kashy et al. (2018), allowing us to examine the trajectories of women and men separately (see the SPSS code in Supporting Information: Table S1). We chose this model since perceived stress is measured as an individual construct, although it also has a dyadic component (Cohen & Wills, 1985). Accordingly, in initial explorations of our data, we found differences in levels of stress between women and men. Women on average reported higher baseline stress compared with men and generally had a higher stress level across the 2‐year period (see Results section). Furthermore, the correlation between partners’ baseline stress was small (Pearson's *r* = 0.18, *p* = 0.007). We therefore estimated fixed and random effects separately for women and men using a dual intercept model with two dummy coded variables (female partner coded 0/1 and male partner coded 0/1). For the random effects, we included a random intercept for each partner, allowing for covariance between them, a random time slope for each partner, and a repeated statement using couple and gender as the subject (PersonID × CoupleID) with the covariance structure specified as autoregressive lag‐1 (AR1). Following the recommendations of Singer and Willett (2003), we examined the temporal trajectories of the data that showed an approximately linear decrease in stress over time with a pivot in women's trajectory at Week 54. Thus, we constructed a linear time slope and modeled the single time point deviating from this slope by including a dummy coded 0/1 break variable of Week 104. Using an ITT approach, this model included all couples (*n* = 230) who filled in the baseline survey, with one exception. Because we needed to treat our sample as distinguishable dyads to test for gender differences between partners, we had to exclude the single same‐sex couple from the dual‐intercept model. We conducted sensitivity analyses including age, education, and income in the model. As these variables were not predictive of change in perceived stress, we left out these parameters in the reporting of results. We calculated Cohen's *d* effect sizes by dividing the estimated effect of each parameter by the pooled SD among men and women separately at baseline.

Across the study period, we found some missing data. Of the 462 participants included at baseline, 86.1% (*n* = 398) were retained after the first year and 79.8% (*n* = 360) were retained at the 2‐year follow‐up (see the flowchart, in the Supporting Information: Figure S1). The rates of missingness were not significantly different between conditions at *χ* ^2^ tests at Week 10, 54, or 104, although there was a trend for more missing values at Week 104 in the control condition than in the intervention condition (*p* = 0.061). Using logistic regression, we did not find missingness at Weeks 10, 54, or 104 to be predicted by baseline stress or gender.

As a total of 13 participants in the intervention group and two participants in the control group reported severe stress on at least one time point, with sum scores ranging from 31 to 39 (*z* > 2.58 equaling the 1% most extreme cases; Field, 2018), we conducted sensitivity analyses for the influence of these participants. These analyses revealed that the intention‐to‐treat (ITT) results were not considerably changed by these 15 severely stressed participants, and we therefore report the results of the ITT analyses.

To investigate the proportion of participants with reliably change in perceived stress, we calculated the reliable change index ($1.96\times \sqrt{{2\times Standard\;\text{Error}}^{2}}$; Jacobson & Truax, 1991) separately for men and women. We conducted two sets of Pearson's *χ* ^2^ tests to compare the proportion of reliable changed participants between conditions after the two checkups and at follow‐up. The first set of *χ* ^2^ tests made use of the full available data by comparing the number of improved (less stressed) participants to the number of stable or more stressed participants (combined categories) between conditions within a gender. The second set of tests omitted the categories of stable participants to compare the number of improved to the number who became more stressed between conditions within a gender.

## RESULTS

### Descriptive statistics of stress in the sample

At baseline, a similar number of women (*n* = 93, 40.1%) and men (*n* = 92, 40.0%) scored above the cutoff for a high stress level, which was nearly twice as many as that seen in the Danish norms (L. Nielsen et al., 2008).^2^ The average baseline stress levels were also elevated compared with Danish norms for both women and men (*M* = 14.94, SD = 6.24 for women; *M* = 13.46, SD = 5.85 for men). We found a gender difference in baseline stress, with women reporting higher baseline stress than men (paired‐samples *t*‐test: mean difference = 1.45, *d* = 0.19, *p* = 0.005), reflecting the normative gender difference. Means and SDs of perceived stress across the seven time points (Years 0–2) are reported in the Supporting Information: Table S2.

### Fixed effects of the MC on perceived stress

**Figure 1:**
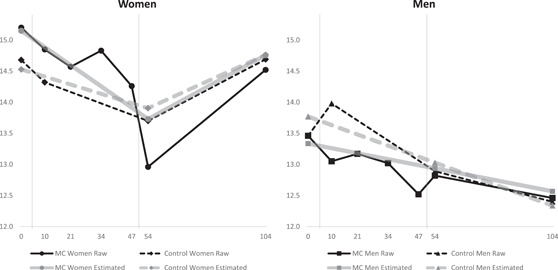
Trajectories based on raw and estimated values of perceived stress in women and men in the intervention and control groups across 2 years. *Y*‐axes are sized to 1 standard deviation. The weeks of the two MCs are marked with vertical lines. Control, The Control Group; MC, Marriage Checkup.

Estimated values and effects of the two MCs are presented in Table 1 and Figure 1. Around half of the total variance in perceived stress was explained by between‐person differences across the study period (intraclass correlations = 0.483 for women and 0.494 for men), with the other half due to changes in stress over time. As expected by the random assignment, no between‐group differences in baseline stress were seen for women or men (*p*s > 0.411). We did not find any between‐group differences in change in perceived stress from baseline to after the second checkup and to follow‐up for women or men (*p*s > 0.309). Within‐group analyses revealed different patterns of change between genders. Women in both groups showed *V‐*shaped trajectories across the study period: They showed a decrease in stress during the year of the intervention, and after the second checkup, this reduction reached a small effect for women in the intervention group (change in PSS = −1.42, *d* = −0.23, *p* = 0.007), but was not significant for women in the control group (change in PSS = −0.62, *d* = −0.10). Between the 1‐ and the 2‐year follow‐up, women in both groups showed an increase in stress and returned to their baseline level. Unlike women, men in both groups showed a steady and nonsignificant decrease in stress across the 2‐year period (estimated change in PSS from Weeks 0 to 104 = −0.77 for the intervention group and −1.44 for the control group).

**Table 1:** Estimated fixed and random effects, effect sizes, and 95% confidence intervals

	Estimate	*SE*	*p*	95% CI	*d*
*Women—Fixed effects*					
Intervention group					
Intercept	15.15	0.53	**<0.001**	[14.10, 16.20]	
Linear time slope (1 = Week 54)	−1.42	0.52	**0.007**	[−2.44, −0.40]	**−0.23**
Deviation from time slope at Week 104	2.35	0.87	**0.007**	[0.65, 4.05]	**0.38**
Differences in the control group					
Difference in intercept	−0.62	0.76	0.411	[−2.11, 0.86]	−0.10
Difference in slope	0.80	0.78	0.309	[−0.74, 2.33]	0.13
Difference in Week 104 deviation	−0.91	1.34	0.495	[−3.53, 1.71]	−0.15
*Men—Fixed effects*					
Intervention group					
Intercept	13.33	0.50	**<0.001**	[12.36, 14.31]	
Linear time slope (1 = Week 54)	−0.40	0.53	0.451	[−1.43, 0.64]	−0.07
Deviation from the time slope at Week 104	0.25	0.88	0.779	[−1.49, 1.98]	0.04
Differences in the control group					
Difference in intercept	0.44	0.70	0.536	[−0.95, 1.82]	0.07
Difference in slope	−0.35	0.78	0.660	[−1.89, 1.20]	−0.06
Difference in Week 104 deviation	0.03	1.35	0.983	[−2.62, 2.67]	0.01
*Random effects ([co‐]variances)*					
Women's intercept	20.80	2.53	**<0.001**	[16.39, 26.39]	4.56
Men's intercept	16.44	2.10	**<0.001**	[12.80, 21.11]	4.05
Covariance of women's and men's intercepts	5.50	1.65	**0.001**	[2.26, 8.73]	
Women's time slope	0.56	0.93	0.543	[0.02, 14.12]	0.75
Men's time slope	0.39	0.89	0.658	[0.00, 32.92]	0.63
Variance of within‐person residuals	21.22	0.94	**<0.001**	[19.46, 23.14]	4.61
Autocorrelation (AR1 ρ)	0.15	0.03	**<0.001**	[0.08, 0.21]	

*Note*: Significant (*p* < 0.05) estimated values and effect sizes are in bold.

Abbreviations: 95% CI, 95% confidence interval; *d*, Cohen's *d*.

We noted that the steeper decrease in stress in women than men in the intervention group (women showed a decrease in stress of 1.02 point more on the PSS than men) after the second checkup was not significant since their confidence intervals were overlapping (women: [−2.44, −0.40]; men: [−1.43, 0.64]).

### Random effects in perceived stress

To facilitate interpretation, we discuss random effects as SDs (taking the square root of the variances reported in Table 1). We found significant variability in baseline stress levels (women: SD = 4.56, men: SD = 4.05). Partners’ baseline stress levels covaried positively, indicating that higher stress in one partner was associated with higher stress in the other partner. The variability around women's slopes and men's slopes was nonsignificant (*p*s > 0.543), indicating that both women and men showed changes in a relatively uniform way and did not differ significantly between persons. The within‐person residuals (AR1 diagonal) showed significant variability (SD = 4.61), indicating a relatively large scattering of scores around each person's trajectory. The autocorrelation was 0.15, indicating that level‐1 residuals in stress at one time point were associated with higher residuals at the following time point.

### Reliable changes in stress after the first and second checkup and at follow‐up

**Figure 2:**
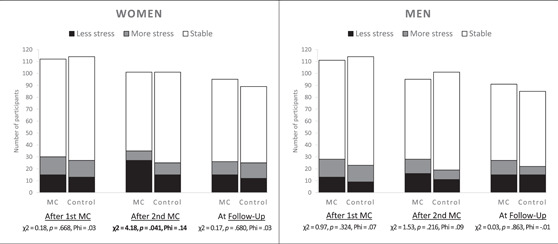
Bar charts of participants (*n*) with reliable changes in perceived stress level. Reliable change required a decrease in the Perceived Stress Scale of 6.67 for men and 6.48 for women from the baseline. *χ* ^2^ tests are based on number improved (less stressed) versus number stable/more stressed between conditions within a gender. Significant results are in bold. Wom., women.

The numbers of participants who experienced reliable improvements or exacerbations in stress are outlined in Figure 2 together with the results of the first set of *χ* ^2^ tests comparing the number of improved participants to the number of stable or more stressed participants. After receiving the first MC, 13.3% of the women and 11.8% of the men showed reliable improvements in perceived stress, which was not significantly different from the control group, in which 11.4% of the women and 7.9% of the men experienced improvements. After the second MC, more women in the intervention group (26.5%) compared with the control group (14.9%) experienced a reliable improvement in perceived stress, showing a small between‐group effect (*φ* = 0.14, *p* = 0.041). For men, the between‐group difference at this point was not significant (17.0% in the MC vs. 10.9% in the control group with reliable improvement). At follow‐up, 15.6% of women and 16.7% of men in the MC group had retained improvement, which was not statistically different from the control group (13.5% of women and 17.6% of men had retained improvement).

Some participants reported elevated stress after the checkups. The percentage who reported reliable elevated stress ranged from 7.8% to 14.6% across gender, time point, and conditions. The second set of *χ* ^2^ tests lost some power (since the large groups of stable participants were omitted), but it did show that the proportions of reliable more stressed participants were not significantly larger or smaller than the proportions of reliably improved participants at any of the three time points in neither women nor men (*p*s > 0.05). Accordingly, there were no between‐group effects in the differences between improved versus more stressed participants (*p*s > 0.153). For the sake of brevity, these nonsignificant results are not reported.

### The proportion of stressed participants

The proportion of highly stressed women in the intervention group shrank from 43.5% (*n* = 50) at baseline to 28.4% (*n* = 29) after the second checkup and 35.4% (*n* = 34) at follow‐up. In the control group, the proportion of highly stressed women remained relatively stable across time from 36.8% (*n* = 43) at baseline to 42.7% (*n* = 38) at follow‐up. For men, the proportion of stressed participants in both groups remained relatively stable, with 38.9% (*n* = 44) and 41.0% (*n* = 48) at baseline to 34.4% (*n* = 31) and 30.6% (*n* = 26) at follow‐up, respectively, for the MC and the control group.

## DISCUSSION

This RCT tested the effect of two annual MCs on individual perceived stress. We did not find between‐group effects on average stress levels at any time point for women or men. Within‐group analyses revealed a *V*‐shaped pattern of change for women in the MC group showing a reduction in stress with a small effect size after the second checkup (*d* = −0.23) and a return to the baseline level at the 2‐year follow‐up. The same pattern of change was seen for women in the control group; however, it was less pronounced and nonsignificant. Men in both groups showed a steady and nonsignificant decrease in average stress across the 2‐year period.

Similar to the within‐group findings, reliable change statistics showed that more women in the MC group (26.5%) than in the control group experienced reliable improvements in stress after the second checkup. No other reliable changes were significant between groups in women or men. Taken together, our findings did not point to either positive or negative main effects of one or two MCs on perceived stress, but did, however, show a small yet transitory relief in stress after the second MC for women.

The current findings contribute toward an otherwise understudied field examining the potential of relationship checkups, and other couple interventions targeting relationship distress, to alleviate symptoms of perceived stress. This field is in its infancy and, to our knowledge, only one previous study has examined and demonstrated positive effects on perceived stress when targeting relationship distress (Roddy, Rhoades, et al., 2020) besides the research field of couple interventions that explicit target dyadic coping with external stressors. Until future studies complement or contradict our findings, the current study highlights the fact that we do not have much proof of individual stress relief produced by a brief couple‐distress‐focused intervention on a population with initial heterogenous stress levels. At the same time, the absence of adverse stress reactions to the MC speaks against the concern of *coachification* and the paradox of health, even though the MC encourages couples towards more proactive care of their relationship health. Although a small number of women and men in the intervention group experienced reliable exacerbation of stress after each checkup, this elevation was probably not induced by the intervention, since a similar pattern was found in the control group. Although the reliable change statistics as well as the random effects indicated some individual variation, the majority of our participants experienced stable (neutral) or relieved stress reactions after receiving the MC. These findings indicate that the proactive approach of recurring checkups on the relationship does not seem to evoke “side effects” in terms of individual stress, but rather holds some potential for relieving individual stress.

### The potential role of gender in stress

We found generally higher stress levels in women than in men (in agreement with normative gender differences, L. Nielsen et al., 2008; The National Board of Health, “Sundhedsstyrelsen”, 2022), a nonsignificantly steeper decrease among women than men, and a larger proportion of women experiencing reliable stress relief after the intervention. A few considerations should be noted regarding these findings. Relationship health might affect individual functioning differently in women and men, which is supported by two recent meta‐analyses (Proulx et al., 2007; Whisman, 2001) pointing to the association between marital quality and individual physical health and mental well‐being to be stronger for women than for men. However, neither of the two meta‐analyses reported on individual stress specifically. Furthermore, this gender difference was found in cross‐sectional studies (*N* = 66 studies), but *not* in longitudinal studies (*N* = 27 studies; Proulx et al., 2007). Likewise, in their RCT, Roddy, Rhoades, et al. (2020) found no gender differences in treatment effects of the two online couple programs on perceived stress. As suggested by Proulx et al. (2007), low levels of relationship functioning might predict individual health, as stress, similarly in women and men *over time*, but women seem to be more affected than men by *concurrent* relationship dysfunction on a day‐to‐day basis. Also, there is an empirically well‐evaluated gender difference in the way in which women and men cope with stress, with women being more likely to turn toward their partner for emotional as well as instrumental support, whereas men are more likely to use coping strategies that do not necessarily involve their partner (Tamres et al., 2002). Moreover, it is confirmed in several studies that women are more likely to seek professional help for relationship issues than men (e.g., Stewart et al., 2016). Correspondingly, in our sample, women were the initiating partner for seeking help (i.e., signing up for the project) in the majority (72.3%, *n* = 167) of the couples. The partner who initiates help‐seeking may also be more ready and motivated for change, which could add to the explanation for the somewhat better gains for the women in our study. However, and against this assumption, we did not find that men were more likely than women to experience elevated stress, nor did men experience increase in stress more frequently than relief in stress.

Finally, instead of attributing the different trends in change between women and men to gender per se, we argue that they could also be attributed to the higher baseline stress characterizing women in our study as well as normatively. As discussed in the following, higher initial stress level entails greater potential for reduction. However, a moderating effect of initial perceived stress level is yet to be investigated in studies of relationship checkups.

### Limitations and future directions

The current study has some methodological limitations that should be noted. First, the sample was characterized by large heterogeneity in levels of individual and couple distress (from high to no level of individual stress and from happy to severely distressed couples). Findings might not generalize to samples with more uniform problem or stress levels. Also, our treatment effect might have been reduced by a floor effect from the large proportion of participants (more than 50%) with initially low stress levels. The impact of improved relationship health on individual stress is weaker when the stress level is low (e.g., when you are not stressed, more social support does not decrease stress much further; Cohen & Wills, 1985; Lakey & Orehek, 2011). Conversely, Roddy, Rhoades, et al. (2020) found that the subsample of participants with initially high stress levels (75.5% of their sample) showed larger reduction in perceived stress (Cohen's *d* = −0.63) than the full sample. Given the potential floor effect in the current study, it is too early to conclude that the MC does not have any relieving effect on individual stress, since it is yet to be tested on samples with more homogeneous disturbing stress levels.

Second, the sample was relatively well educated and had higher income than the average Danish couple, both of which limit the generalizability to couples with lower socioeconomic status. Findings of Roddy, Rhoades, et al. (2020) support the assumption that couples with lower education (16.1% had a bachelor's degree or higher) and low income (42% were at or below the U.S. federal poverty line) would experience stress reduction after couple interventions aimed at relationship functioning.

Third, we measured perceived stress longitudinally using self‐report evaluations. This does not capture momentary aspects of exacerbated stress potentially induced by the intervention (i.e., entering the therapist office, dealing with conflict during the session) or psychophysiological stress symptoms that could also be affected by changes in couple functioning (including cortisol regulation, e.g., Ditzen et al., 2011; Slatcher et al., 2015; Stanton et al., 2019). Physiological measures such as salvatory cortisol or heart rate may be better fitted for the purpose of intensive assessment of fluctuations in stress.

Fourth, we had to omit one same‐sex couple from the dual‐intercept model to allow an analysis approach for distinguishable dyads when testing gender differences between partners. To our knowledge, there are currently no data analytic approaches available that allow combining distinguishable and indistinguishable dyads. Future methodological improvements in dyadic analyses will hopefully accommodate more diverse dyads.

Finally, we did not conduct comprehensive analyses of possible moderators of change. From our findings, it seems relevant to further test moderators as the initial baseline stress level and different sources of stress (i.e., external or internal to the relationship). Also, it remains unknown whether changes in stress are mediated by, for example, changes in relationship functioning, as was found for depression outcomes (Gray et al., 2020).

## CONCLUSION

This RCT contributes to a better understanding of the MC, with findings indicating that its proactive strategy for early detection and treatment of relationship health issues does not seem to evoke adverse “side effects” of exacerbating stress levels in a sample with initially heterogeneous perceived stress levels. Rather, the recurrence of MCs seems to hold potential for relieving stress symptoms, with some indications of greater yet more transitory relief for women than for men.

## Supporting information

Supplementary information.

## ENDNOTES

^1^
These cut‐offs are not clinically defined. In L. Nielsen et al. (2008), a high stress level was defined as a sum score of ≥17 for women and ≥15 for men on the Perceived Stress Scale that ranges from 0 to 40. In the National Board of Health (2022), this cut‐off was 18 for both genders.

^2^
Since our baseline survey was conducted in 2013 through 2014, we compare the responses to the Danish norms of 2008 instead of the recent national records from 2021 to avoid the bias of a potential cohort effect of, for instance, the COVID‐19 pandemic.
