# Supplementary material for: Randomized controlled trial of the Marriage Checkup: Stress outcomes
Source: J Marital Fam Ther. 2022 Dec 16;49(1):242–59. doi: 10.1111/jmft.12620 (PMC10108012; doi:10.1111/jmft.12620)
Supplement: Supplementary file 1 — Supplementary information. [file JMFT-49-242-s001.docx]

# **Supplemental Materials**

Leth-Nissen, A. B., Fentz, H., N., Trillingsgaard, T. L., & Stadler, G. (2022). *Randomized Controlled Trial of the Marriage Checkup: Stress Outcomes.* [Manuscript submitted for publication]. Department of Psychology and Behavioural Sciences, Aarhus University.

**Figure S1**

*Consort Diagram*

*
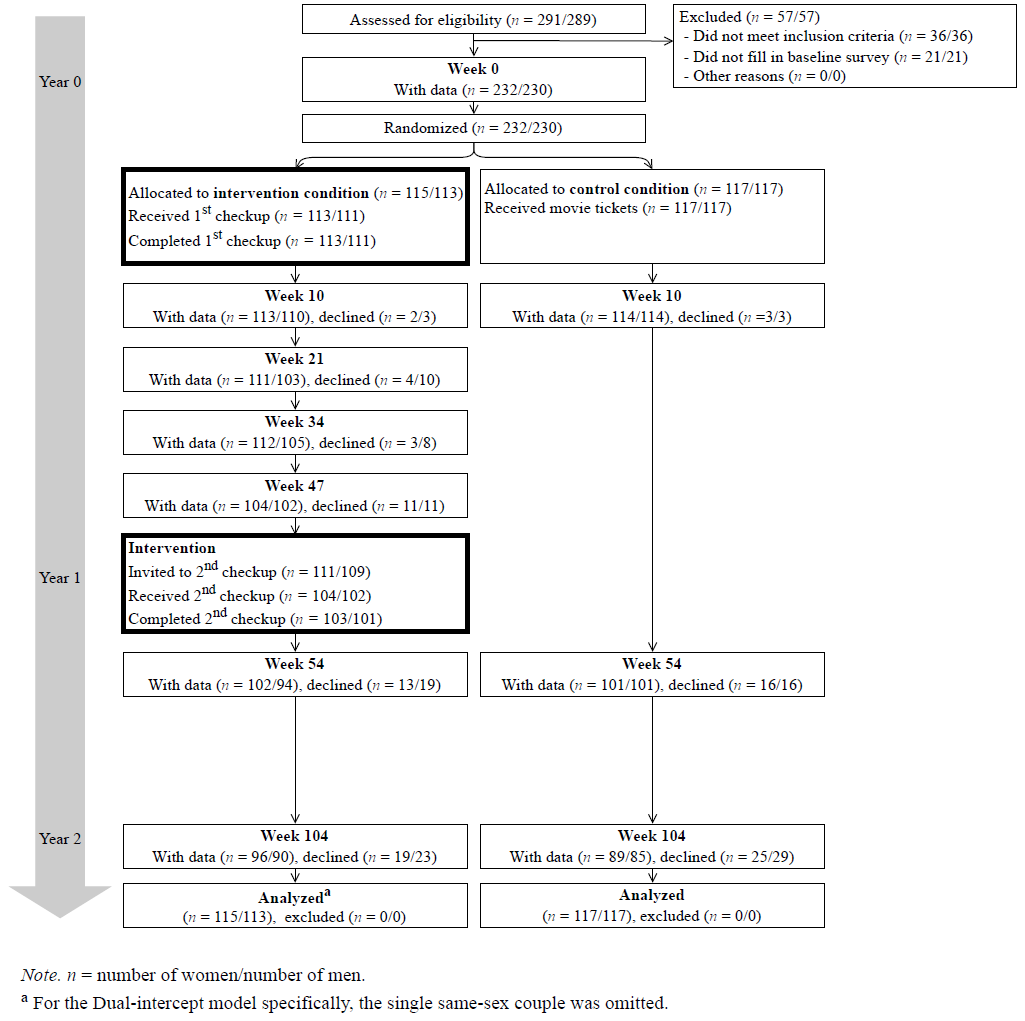
*

| **Table S1**  *SPSS Code for the Dual-Intercept Multilevel Model (N = 460 Participants)* |
| --- |
| MIXED PSS WITH Man01 Wom01 Condition Time Week104as01  /FIXED =  Man01  Man01*Condition  Man01*Time  Man01*Time*Condition  Man01*Week104as01  Man01*Week104as01*Condition  Wom01  Wom01*Condition  Wom01*Time  Wom01*Time*Condition  Wom01*Week104as01  Wom01*Week104as01*Condition  \| NOINT  /PRINT = G SOLUTION TESTCOV CORB  /METHOD = REML  /RANDOM Man01 Wom01 \| SUBJECT(CoupleID) COVTYPE(UN)  /RANDOM Man01*Time Wom01*Time \| SUBJECT(CoupleID) COVTYPE(VC)  /REPEATED = Time \| SUBJECT(PersonID*CoupleID) COVTYPE(AR1). |
| \| **Parameter** \| **Coding** \| \| --- \| --- \| \| PSS \| Sum score of perceived stress ranging from 0 to 40. \| \| Man01 \| 0 = not man,  1 = man \| \| Wom01 \| 0 = not woman,  1 = woman \| \| Condition \| 0 = Marriage Checkup group,  1 = Control group \| \| Time \| 0 = Week 0  0.04 = Week 10  0.39 = Week 21  0.63 = Week 34  0.87 = Week 47  1 = Week 54  1.93 = Week 104 \| \| Week104as01 \| 0 = Week 0  0 = Week 10  0 = Week 21  0 = Week 34  0 = Week 47  0 = Week 54  1 = Week 104 \| \| CoupleID \| Couple identification (same for male and female partner) \| \| PersonID \| Personal identification (unique per participant) \| |
| *Note*. The single same-sex couple was omitted for this analysis. |

| **Table S2**  *Descriptive Statistics for Perceived Stress (N = 462 Participants)* | | | | | | | | | | | | |
| --- | --- | --- | --- | --- | --- | --- | --- | --- | --- | --- | --- | --- |
|  | Intervention group | | | | | | Control group | | | | | |
|  | Women (*n* = 115) | | | Men (*n* = 113) | | | Women (*n* = 117) | | | Men (*n* = 117) | | |
| Week | Mean | *SD* | *n* | Mean | *SD* | *n* | Mean | *SD* | *n* | Mean | *SD* | *n* |
| 0 | 15.20 | 6.21 | 115 | 13.46 | 6.28 | 113 | 14.68 | 6.29 | 117 | 13.46 | 5.42 | 117 |
| 10 | 14.85 | 6.67 | 113 | 13.05 | 6.61 | 110 | 14.32 | 6.62 | 114 | 13.98 | 5.39 | 114 |
| 21 | 14.57 | 6.68 | 111 | 13.17 | 6.24 | 103 | - | - | - | - | - | - |
| 34 | 14.83 | 6.75 | 112 | 13.02 | 6.60 | 105 | - | - | - | - | - | - |
| 47 | 14.26 | 6.94 | 104 | 12.52 | 6.03 | 102 | - | - | - | - | - | - |
| 54 | 12.96 | 6.30 | 102 | 12.82 | 6.68 | 94 | 13.70 | 6.84 | 101 | 12.89 | 5.26 | 101 |
| 104 | 14.52 | 6.56 | 96 | 12.46 | 6.26 | 90 | 14.69 | 6.46 | 89 | 12.41 | 5.74 | 85 |
| *Note.* The two Marriage Checkups were conducted at Week 7 and 51. | | | | | | | | | | | | |
